# Targeting Prolyl 4-Hydroxylase Subunit Beta (P4HB) in Cancer: New Roads to Travel

**DOI:** 10.14336/AD.2023.1126

**Published:** 2023-11-26

**Authors:** Dechao Feng, Jie Wang, Dengxiong Li, Ruicheng Wu, Zhouting Tuo, Qingxin Yu, Luxia Ye, Akira Miyamoto, Koo Han Yoo, Cheng Wang, Yuanzhi Cheng, Xing Ye, Chi Zhang, Wuran Wei

**Affiliations:** ^1^Department of Urology, Institute of Urology, West China Hospital, Sichuan University, Chengdu, China.; ^2^Department of Rehabilitation, The Affiliated Hospital of Southwest Medical University, Luzhou, China.; ^3^Chengdu Basebio Company, China.; ^4^Department of Urology, The Second Affiliated Hospital of Anhui Medical University, Hefei, China.; ^5^Ningbo Diagnostic Pathology Center, Ningbo, Zhejiang, China.; ^6^Department of Public Research Platform, Taizhou Hospital of Zhejiang Province Affiliated to Wenzhou Medical University, Linhai, China.; ^7^Department of Rehabilitation, West Kyushu University, Japan.; ^8^Department of Urology, Kyung Hee University, South Korea.; ^9^Cedars-Sinai Medical Center, Los Angeles, CA, USA.

**Keywords:** P4HB, pan-cancer analysis, drug sensitivity, endoplasmic reticulum response

## Abstract

Prolyl 4-hydroxylase subunit beta (P4HB) can catalyze the formation, breakage and rearrangement of disulfide bonds through two thioredoxin domains, which is important for the maintenance of oxidizing environment in endoplasmic reticulum. Recently, P4HB has been demonstrated its oncogenic role of tumorigenesis and development in cancers. Therefore, we comprehensively deciphered P4HB in human cancer from various aspects, including pan-cancer analysis and narrative summary. We also provided some possible interacted molecules and the top 10 predicted drugs targeting P4HB to contribute to future research. We proposed that P4HB was a potential target and brought new therapeutic opportunities for cancer patients.

Prolyl 4-hydroxylase subunit beta (P4HB), also known as protein disulfide-isomerase, glutathione-insulin transhydrogenase, or cellular thyroid hormone-binding protein, encodes the beta subunit of prolyl 4-hydroxylase. This enzyme is responsible for hydroxylating prolyl residues in preprocollagen [1]. P4HB is located on 17q25.3 and can catalyze the formation, breakage and rearrangement of disulfide bonds through two thioredoxin domains [1]. Moreover, P4HB is an essential component of the endoplasmic reticulum (ER). ER is a major organelle involved in intracellular calcium control and protein folding and is essential for cellular homeostasis. The ER provides an oxidizing environment that promotes nascent protein folding and disulfide bond production and is maintained by redox sensor glutathione (GSH) or oxidized glutathione (GSSG). P4HB oxidizes reduced polypeptides and provides their electrons to endoplasmic reticulum oxidase 1 (ERO1), a major mediator of disulfide bond formation in the ER [2]. In a diseased condition, misfolded and unfolded proteins may accumulate, leading to the enlargement of the ER and activation of the ER stress response. This process in turn activates the unfolded protein response. On the one hand, this process can favor cell survival, and on the other hand, in the presence of prolonged ER stress, it can also trigger cell apoptosis [2-4]. Thus, P4HB can function as an important molecular chaperone of ERO1 to regulate the ER homeostasis. In the last two decades, researchers have been attracted by its important role in a wider range of cancers, especially since 2020. Considering these facts, this perspective presents a comprehensive summary of the effect of P4HB on human cancers through bioinformatic analysis and current experimental evidence. Future treatments for these disorders may be built on the knowledge provided by the current findings.

## PAN-CANCER ANALYSIS OF P4HB

**Figure 1.**
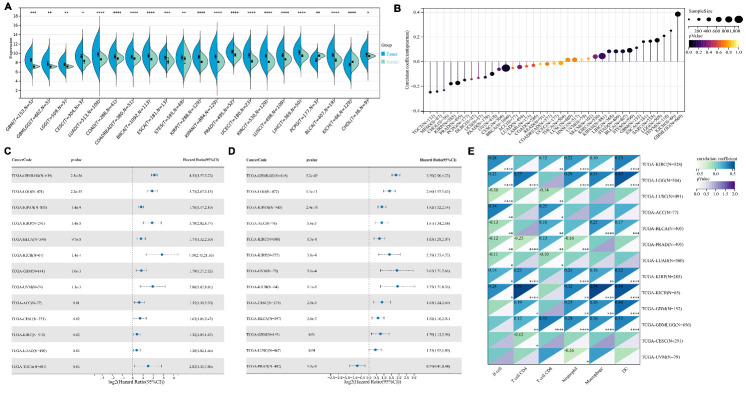
Pan-cancer analysis of P4HB.

Using the methods of our previous studies [5-8], we initially used pan-cancer analysis to explore the association between P4HB and cancers. We found that P4HB was almost expressed higher in tumor samples than in normal samples and was closely related to age in many cancers (Fig. 1A-B), which is consistent with the fact that age is risk factor of a variety of cancers [9-13]. Moreover, it was also highly related to overall survival (OS) (Fig. 1C) and progression-free survival (Fig. 1D) in various cancers with statistical significance. Among these cancers, we observed that P4HB was significantly associated with the prognosis of patients with urinary tumors (adrenocortical carcinoma (ACC), kidney renal papillary cell carcinoma (KIRP), kidney renal clear cell carcinoma (KIRC), kidney chromophobe (KICH), pan-kidney cohort (KICH+KIRC+KIRP), bladder urothelial carcinoma (BLCA) and prostate adenocarcinoma (PRAD), lung cancer (lung adenocarcinoma (LUAD) and lung squamous cell carcinoma (LUSC), gliomas (glioblastoma multiforme (GBM), glioma (GBMLGG) and brain lower grade glioma (LGG), cervical squamous cell carcinoma and endocervical adenocarcinoma (CESC) and uveal melanoma (UVM) with significant differential expression between tumor and normal samples. Additionally, we found that P4HB expression was significantly related to tumor-infiltrating cells in patients with renal cancer and glioma (Fig. 1E). In terms of immune regulatory genes, we detected that P4HB showed greater correlation (absolute value of r > 0.4) with them for patients with GBM, UVM, renal cancer, ACC and PRAD, especially for renal cancer, GBM and UVM (Supplementary Table 1). Similar results were observed for the relationship between P4HB and immune checkpoints (Supplementary Table 2).

## EFFECT OF P4HB ON HUMAN CANCER

### P4HB and urinary tumors

**Figure 2.**
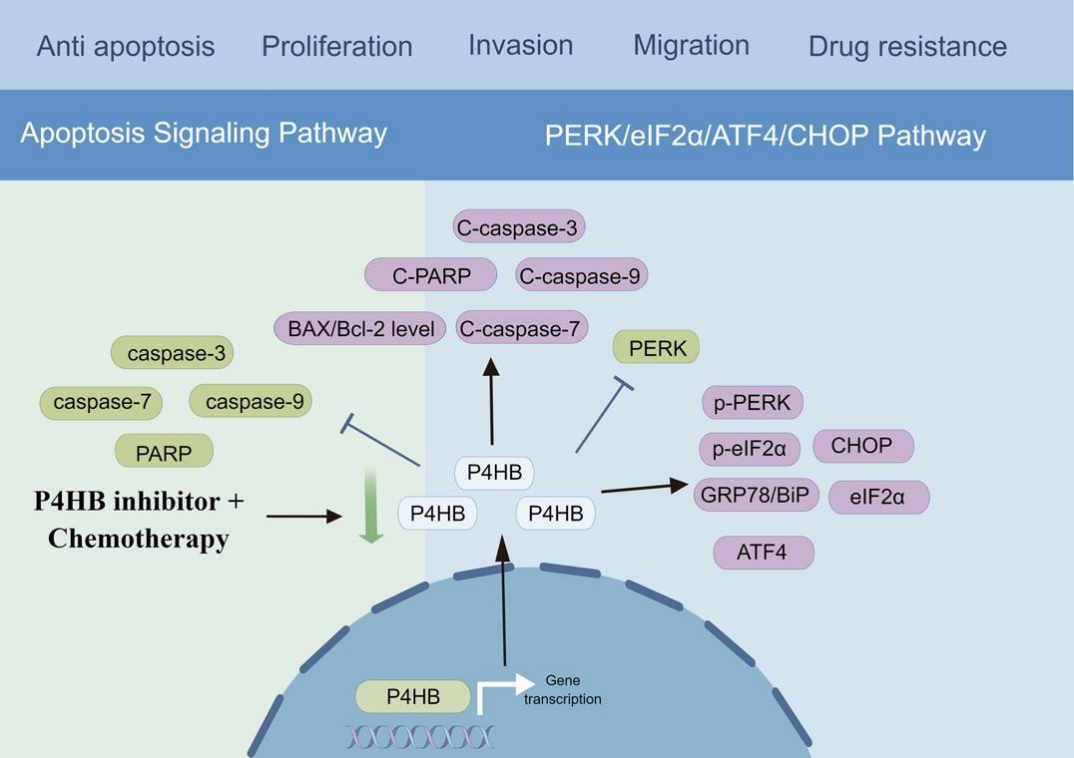
The underlying mechanism of P4HB in bladder cancer.

BLCA is the 10th most diagnosed cancer worldwide, with more than 500 thousand new cases and 200 thousand fatalities expected in 2019 [14, 15]. The global incident cases of BLCA in 2019 were 123.34% more than in 1990 [16]. BLCA is still a challenging cancer to cure due to its high rate of recurrence, need for close monitoring, and invasive methods of diagnosis and therapy [17]. Our previous study showed that P4HB was highly expressed in BLCA tissues than in paired normal tissues [18]. Patients with high P4HB expression were significantly associated with a worse OS and disease-free survival (DFS) [18, 19]. In vitro experiments showed that the suppression of P4HB could reduce the proliferative capacity of BLCA cells, which was consistent to the finding of Lyu et al. [19]. Mechanically, in our previous study, we utilized the TCGA database to conduct Gene Set Enrichment Analysis (GSEA) on the high P4HB expression cohort to identify enriched biological pathways. We found that high P4HB group was mainly enriched in amino sugar and nucleotide sugar metabolism, glycan biosynthesis, glycosphingolipid biosynthesis, galactose metabolism, other glycan degradation, steroid biosynthesis, and biosynthesis of unsaturated fatty acids, which indicated P4HB may influence nutrient metabolism in BLCA. In addition, we observed an upregulation of ER stress pathway proteins, including 78-kilodalton glucose regulated protein (GRP78/BiP), p-PERK, eIF2α, p-eIF2α and ATF4 after treating BLCA cells with bacitracin, a kind of P4HB inhibitor, which suggested that the inhibition of P4HB activated the PERK/eIF2α/ATF4/CHOP signaling pathway [18] (Fig. 2). Additionally, the downregulation of P4HB expression statistically enhanced the sensitivity of BLCA cells to gemcitabine treatment by activating the same pathways (Fig. 2). Furthermore, P4HB also served as a core role in the autophagy regulatory mechanisms of BLCA, warranting further investigation and exploration [19].

As the population ages over the coming decades, PRAD is one of the most common cancers in the urinary system, placing additional strain on the healthcare system [20-26]. Consistent with our pan-cancer analysis, our previous studies[8, 27] found that P4HB expression was higher in tumor samples than in normal samples and P4HB downregulation could significantly inhibit the cell proliferation of six PRAD cell lines, including LNCap, C4-2, C4-2B, PC3, DU145 and 22RV1 cells. In addition, renal cancer is also a common urinary tumor which ranks the 9th most common type of cancer in males and accounts for 4.2% of novel diagnoses [28-30]. Several studies used bioinformatic analysis to indicate that P4HB was significantly associated with prognosis for KIRC and KIRP patients [31-36], which was consistent with our finding in this study.

### P4HB and female tumors

In ovarian cancer, P4HB was also expressed higher in tumor tissues compared to adjacent tissues [37]. Similarly, the sensitivity of ovarian cancer cells to cisplatin and phytochemicals might be regulated by the expression of P4HB [38]. In terms of endometrial carcinoma, Colas et al. [39] discovered differential expression of P4HB in tumor and normal tissues of endometrial carcinoma, identifying it as the most specific and sensitive marker for this cancer type.

Breast cancer (BRCA) is the predominant malignancy among women globally, posing a significant threat to both their physical and mental well-being [40]. In 2020, it is estimated that 2,261,419 new cases of BRCA and 684,996 deaths from BRCA [40]. In the level of mRNA expression, P4HB was found to be upregulated in BRCA tissues and BRCA cell lines compared to normal tissues and cell lines [41]. In the level of protein, Zhang et al. found P4HB was upregulated in HER-2/neu-positive breast tumors [42]. In addition, Gwark et al. [43] detected the plasma proteome of 51 non-metastatic BRCA patients who underwent neoadjuvant chemotherapy (NCT). They found that P4HB had higher plasma concentrations in the non-pathological complete response (pCR) group [43]. Meanwhile, low serum P4HB level was significantly associated with better DFS and OS [43]. These findings suggested that P4HB could serve as a potential biomarker for predicting the prognosis of BRCA patients and their response to NCT to a certain extent. Mechanistically, Yang et al. [41] found that P4HB could directly interact with collagen type X alpha 1 (COL10A1) through co-immunoprecipitation (Co-IP) assay. COL10A1 was overexpressed in BRCA patients and cell lines. Functionally, high expression of COL10A1 amplified the proliferation and metastatic capabilities of BRCA cells, while suppression of COL10A1 expression hindered the progression of BRCA. The downregulation of P4HB could suppress the promoting effects exerted by overexpressing COL10A1 on the proliferation, migration, and invasion of BRCA cells. Conversely, upregulation of P4HB enhances BRCA cell proliferation, clone-forming capacity, migration and invasion [41]. This indicated that COL10A1 could serve as an oncogene and function through upregulating P4HB expression.

### P4HB and digestive tumors

**Figure 3.**
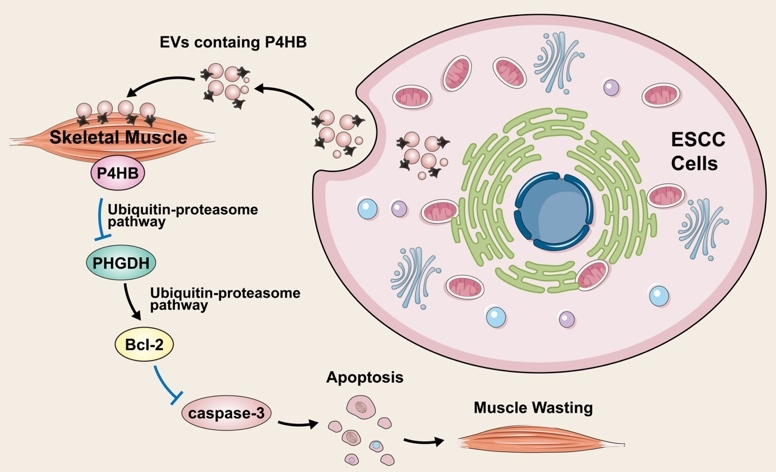
The underlying mechanism of P4HB in oesophageal squamous cell carcinoma.

In 2021, Gao et al. [44] developed an esophageal squamous cell carcinoma (ESCC)-induced cachexia mouse model using human xenograft ESCC cell lines. Then, they observed that extracellular vesicles secreted by ESCC cells, containing P4HB, induced apoptosis in C2C12 myoblasts by upregulating the expression of cleaved PARP, caspase-3, and caspase-8. Mechanically, using Co-IP assay and glutathione S-transferase affinity isolation assay, they found P4HB could directly interact with phosphoglycerate dehydrogenase (PHGDH). In addition, they revealed that wild-type P4HB and the first domain (a sequence spanning amino acids 1-138) retained their interaction with PHGDH. Considering that redox-active domain a (amino acids 1-138) is known to facilitate redox reactions [45], it is plausible that this domain plays a crucial role in enabling their interaction and regulation. Furthermore, they found the overexpression of P4HB could notably accelerate PHGDH degradation using cycloheximide pulse-chase assay, resulting in the downregulation of Bcl-2 expression and ultimately inducing apoptosis. Conversely, reducing P4HB levels prolonged the half-life of PHGDH. Western blot analysis unveiled that PHGDH degradation was facilitated via ubiquitin-dependent proteolytic pathway. Additionally, the observed reversal of these phenomena by the P4HB inhibitor, CCF642, provides compelling evidence of the significant involvement of P4HB in the progression of ESCC b via regulating PHGDH/Bcl-2/caspase-3 pathway (Fig. 3).

In gastric cancer (GC), P4HB was found to exhibit a strong correlation with Hypoxia Inducible Factor-1α (HIF1α). Zhang et al. [46] found the overexpression of HIF1α and P4HB was associated with worse DFS and OS of GC patients. In addition, the higher level of HIF1α and P4HB had a significant correlation with higher TNM staging and peritoneum cavity metastasis [46]. Similarly, another study compared the differentially expressed genes between GC cells and HIF-1α-knockdown GC cells. They identified P4HB as a hub gene via centrality analysis and Molecular Complex Detection (MCODE) module analysis [47]. Furthermore, the overexpression of P4HB could partially reverse the weakened invasive and migratory capabilities of GC cells caused by the inhibition of HIF-1α, which suggested P4HB played an important role in the regulatory network of HIF-1α [47].

**Figure 4.**
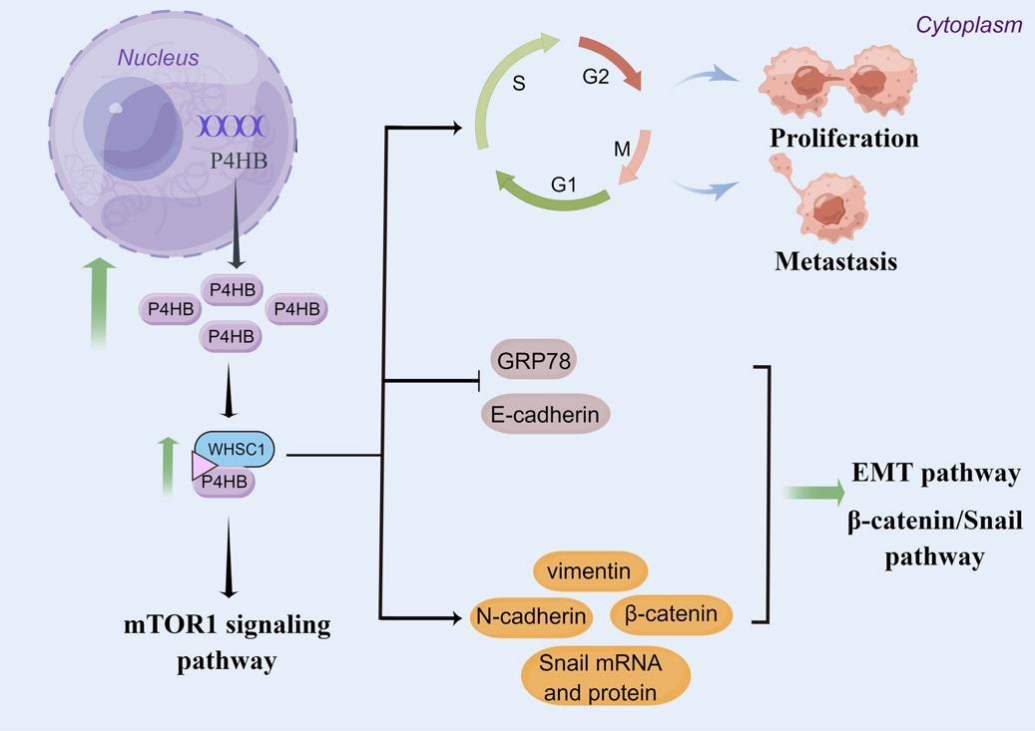
The underlying mechanism of P4HB in hepatocellular carcinoma.

In hepatocellular carcinoma (HCC), P4HB was overexpressed in HCC tissues and cell lines and higher P4HB levels were associated with advanced disease and worse prognosis [48, 49]. In vivo experiments, the overexpression of P4HB could promote HCC cell growth, migration, invasion and epithelial-to-mesenchymal transition (EMT), while P4HB knockdown could inhibit HCC tumorigenesis [48, 50]. In addition, P4HB was also found to be associated with liver cancer chemoresistance. Ma et al. [50] compared some mRNAs and proteins between HepG2/adriamycin (ADR) cells and HepG2 cells. They found E-cadherin mRNA and protein expression levels was decreased while P4HB, N-cadherin and vimentin mRNA and protein expression levels were increased in HepG2/ADR cells [50]. Furthermore, P4HB knockdown resulted in a notable decline in cell viability, invasion and migration capabilities in HepG2/ADR cells. Additionally, the knockdown of P4HB resulted in increased mRNA and protein expression levels of E-cadherin. Conversely, the levels of N-cadherin, vimentin, total β-catenin, nuclear β-catenin and Snail mRNA and protein were significantly decreased [50]. These results provided evidences that the modulation of P4HB in liver cancer chemoresistance is influenced by EMT and β-catenin/Snail pathway. Mechanistically, P4HB was found to be negatively correlated with GRP78 [48]. GRP78 stands as the predominant ER chaperone and serves as a pivotal regulator of the mammalian unfolded protein response [51]. Suppression of GRP78 expression was found to enhance the migratory potential of colon cancer and HCC cells by inducing EMT [52, 53]. Xia et al. [48] found that the overexpression of P4HB could downregulate GRP78, thereby promoting the progression of HCC (Fig. 4). Moreover, Dai et al.[54] found Wolf-Hirschhorn syndrome candidate gene-1 (WHSC1) was significantly increased in HCC tissues and cell lines. The high expression of WHSC1 was associated with adverse clinicopathological characteristics. Using immune-precipitation/mass spectrum analysis, they identified P4HB as the binding partner of WHSC1. WHSC1 demonstrated an interaction with P4HB, leading to the stimulation of P4HB expression and consequent activation of mTOR1 signaling (Fig. 4).

In colon cancer, the expression of P4HB was higher in tumor tissue compared to normal tissue [55]. In vitro experiments suggested that the knockdown of P4HB could increase the apoptosis of human HT29 cells [55]. Mechanistically this may be achieved through inhibiting of the signal transducer and activator of transcription 3 (STAT3) signaling pathway and inducing accumulation of reactive oxygen species (ROS). Attenuating the accumulation of ROS could nullify the increased cell apoptosis triggered by P4HB depletion [55]. Additionally, there is limited literature available regarding the role of P4HB in pancreatic tumors. Güven [56] identified 19 hub genes including P4HB between pancreatic ductal adenocarcinoma (PDAC) tissues and adjacent normal tissues through Network Analyst web tool founded on protein-protein interaction network through the STRING database. In addition, Zhu et al. [57] identified differentially expressed proteins between solid pseudopapillary tumor of the pancreas (SPTP) tissues and matched normal pancreas tissues and found P4HB was downregulated in SPTP.

### P4HB and gliomas

Diffuse gliomas contain oligodendrogliomas, astrocytomas, oligoastrocytomas and GBM, which are the predominant type of primary central nervous system (CNS) tumors, and result in the highest mortality among all brain and CNS tumors [58, 59]. P4HB was found to upregulated in glioma tissues compared to normal tissues [60, 61]. Glioma patients with higher levels of P4HB had a worse prognosis [60-62]. In addition, the correlation analysis revealed a positive association between P4HB expression and interferon-gamma-inducible protein 30 (IFI30) expression [60], Ki-67 and high frequency of the TP53 mutation[61]. Notably, P4HB has been proved to be involved in the development of chemoresistance in gliomas. Sun et al. [63] discovered that P4HB was overexpressed in temozolomide (TMZ) resistant GBM cells and vivo xenografts. Clinically, they observed an up-regulation of P4HB in recurrent GBM patients who initially showed responsiveness to TMZ but subsequently developed acquired resistance, in comparison to treatment-naive tumors [63]. In vitro and vivo experiments, P4HB knockdown and inhibition could sensitize TMZ resistant GBM cells. The overexpression of P4HB could restore this TMZ-resistance. Mechanistically, the inhibition of P4HB disrupted its protective function and enhanced the sensitivity of glioma cells to TMZ by activating the protein kinase R-like endoplasmic reticulum kinase (PERK) arm of the ER stress response [63]. Additionally, miRNA-210 could downregulate P4HB thereby resulting in a reduction in TMZ-resistance [64].

**Figure 5.**
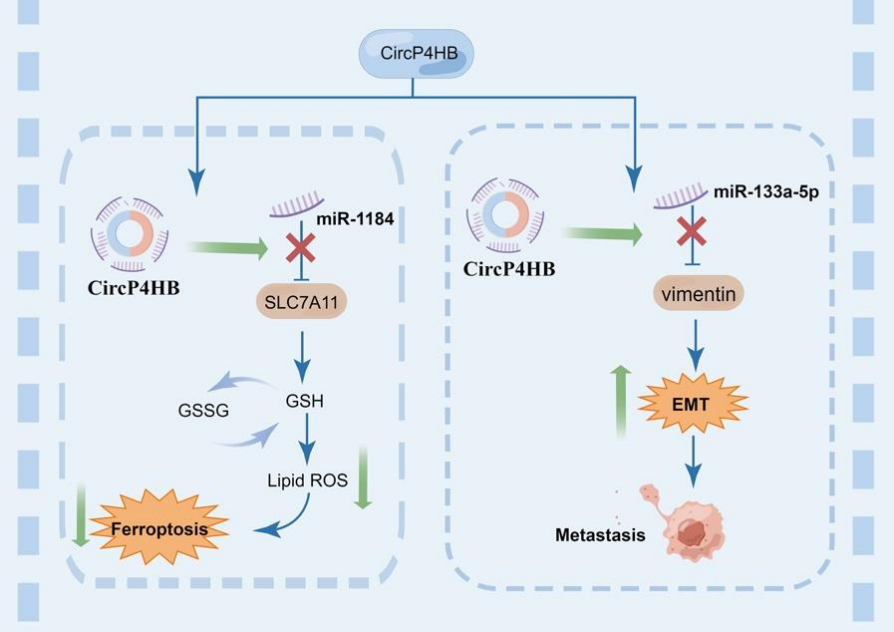
The underlying mechanism of P4HB in lung cancer.

### P4HB and lung cancer

Lung cancer is one of the most common malignant tumors globally, characterized by high metastatic potential and mortality rates [40, 65]. Lung cancer is mainly classified into two primary categories: non-small-cell lung cancer (NSCLC), which accounts for approximately 85% of cases, and small-cell lung cancer, which represents about 15% [66]. Among NSCLC subtypes, LUAD is the most prevalent histological subtype, accounting for approximately 60% of cases [67]. Studies on P4HB in lung cancer mainly focused on bioinformatics analysis. P4HB was overexpressed in NSCLC tissues compared to adjacent normal tissues [68-71]. High expression of P4HB was associated with worse prognosis of LUAD patients [71]. Hua et al [70]. and Liu et al [71]. identified some hub genes including P4HB in LUAD through bioinformatic analysis. Interestingly, two studies explored the underlying carcinogenic mechanisms of circP4HB in LUAD and found circP4HB was upregulated in patient-derived NSCLC tissue versus paired healthy samples. The higher circP4HB levels were associated with metastatic disease and poorer survival [72, 73]. Mechanistically, on the one hand, pan et al. [73] found that circP4HB could trigger GSH synthesis, thereby protecting LUAD cells from erastin-induced ferroptosis. CircP4HB potentially operated as a competing endogenous RNA, exerting influence on miR-1184 to modulate the expression of Solute carrier family 7 member 11 (SLC7A11). Its role involved the inhibition of ferroptosis by orchestrating the miR-1184/SLC7A11-mediated synthesis of GSH. Notably, in vivo experiments, the overexpression of circP4HB facilitated tumor growth and suppressed the occurrence of ferroptosis. On the other hand, circP4HB could stimulate the expression of miR-133a-5p to upregulate vimentin, leading to EMT and metastasis [72] (Fig. 5).

### P4HB and other tumors

Acute myeloid leukemia is an extremely aggressive hematological malignancy, exhibiting a wide range of molecular abnormalities and the accumulation of immature myeloid progenitors in both the bone marrow and peripheral blood [74, 75]. Fu et al. [76] used eight autophagy-related genes to identify an autophagy-related signature including P4HB and found it could be used as an independent prognostic predictor for acute myeloid leukemia patients.

## PERSPECTIVE

**Figure 6.**
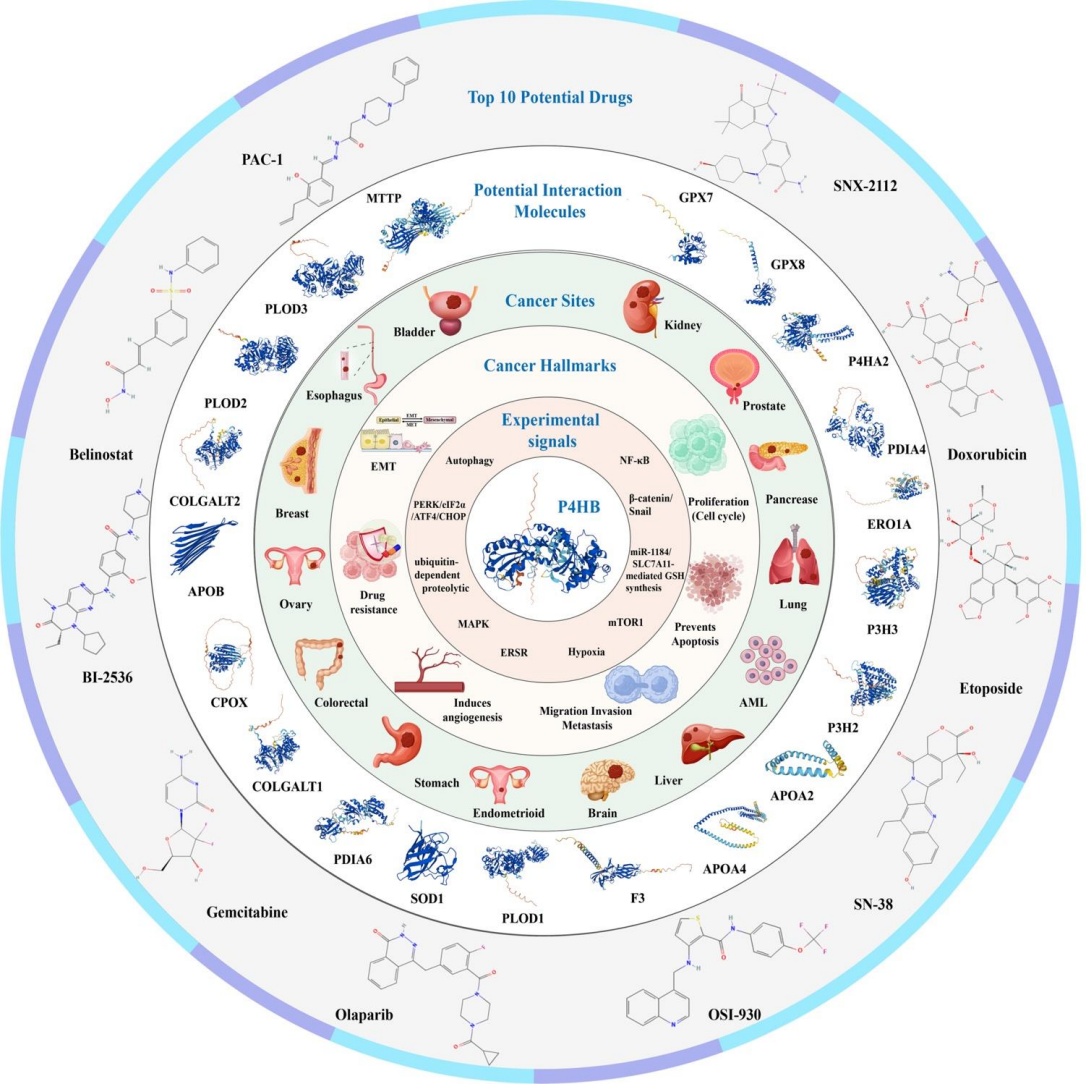
Summary of the findings in this perspective.

Current evidence indicates that although P4HB have been demonstrated to be higher expressed in almost all of tumor samples than in normal samples and it can significantly influence some phenotypes of cancers, like cell proliferation, invasion, migration and even drug sensitivity. However, the specific mechanisms underlying the effect of P4HB on tumors are still superficial in many cancers. For example, our previous studies and pan-cancer analysis showed that P4HB was closely related to urinary tumors, especially for renal cancer, but more in-depth mechanism were less, which in turn demonstrated the role of P4HB as a potentially therapeutic target. GeneMANIA is a powerful online database and tool used for analyzing the functions, interactions, and associations of genes and proteins. It aids researchers in understanding gene functionality and their roles in biological processes. This platform integrates extensive bioinformatics data, including gene expression data, protein interactions, co-expression, gene regulation, and more, enabling prediction, analysis, and visualization of relationships between genes [77]. Using GeneMANIA server [77], we found that P4HB was predicted to interact with several molecules, including MTTP, PLOD3, PLOD2, COLGALT2, APOB, CPOX, COLGALT1, PDIA6, SOD1, PLOD1, F3, APOA4, APOA2, P3H2, P3H3, ERO1A, PDIA4, P4HA2, GPX8 and GPX7. PDIA6 and PDIA4 are the members of the disulfide isomerase (PDI) family of ER proteins that catalyze protein folding and thiol-disulfide interchange reactions [78, 79]. These chaperone proteins are crucial for protein folding and modification. Their interaction with P4HB may orchestrate proper folding of oncogenic proteins, affecting signaling pathways vital for cancer cell proliferation and survival. Recent researches indicated that PDIA6 was upregulated in human cancers such as liver cancer and bladder cancer. Moreover, knocking down PDIA6 expression can reduce the proliferation and invasion of bladder cancer cells [80, 81]. Similarly, PDIA4 was also found to upregulate in ESCC, glioma and so on. PDIA4 was reported to inhibit PRAD cell apoptosis and drove docetaxel resistance via activating the Akt-signaling pathway [82]. Additionally, in ovarian cancer, PDIA4 could activate PI3K/AKT signaling to promote the growth of ovarian cancer cells through mediating miR-378a-3p [83]. Based on these findings, investigating the role of these interactions among P4HB, PDIA4 and PDIA6 in driving aggressive tumor phenotypes, such as proliferation, invasion and metastasis, could uncover potential targets for anti-metastatic therapies. GPX7 and GPX8 are the members of glutathione peroxidase (GPXs) family, which mainly serve as antioxidant enzymes to reduce various ROS such as hydrogen peroxide and lipid peroxides [84]. Given P4HB's role in protein folding and GPX7/GPX8's function in scavenging ROS, their interaction could influence the redox environment crucial for cancer cell survival and proliferation. For example, the interaction between P4HB and GPX7/GPX8 might impact cancer cell responses to oxidative stress. This interaction could affect the efficiency of ROS scavenging and thus influence the cellular response to oxidative damage induced by factors like chemotherapy or radiation therapy, thereby resulting in chemotherapy resistance and radiotherapy resistance. Manipulating these interactions might provide novel avenues for targeted therapies aimed at disrupting redox balance or sensitizing tumor cells to treatment. In brief, in future, in vitro and in vivo experiments are still needed to verify the practical significance of these predicted interactions for tumor development and therapy. Moreover, based on the GSCALite [85], we predicted the top 10 potential drugs targeting P4HB at pan-cancer level, which might contribute to target drug development and clinical application in the future. Figure 6 presents summary of the findings in this perspective.

## CONCLUSIONS

In this perspective, we used bioinformatic analysis and current experimental evidence to demonstrate that P4HB might serve as a potent target in various cancers. We found that P4HB was upregulated in most tumors and was associated with poor prognosis. In terms of immune regulatory genes, we found P4HB showed greater correlation with them for patients with GBM, UVM, renal cancer, ACC and PRAD, especially for renal cancer, GBM and UVM. Through narrative review, we summarized the signaling pathways and cancer hallmarks involved in P4HB in human cancers. Based on GeneMANIA database, we found that P4HB was predicted to interact with several molecules, including MTTP, PLOD3, PLOD2, COLGALT2, APOB, CPOX, COLGALT1, PDIA6, SOD1, PLOD1, F3, APOA4, APOA2, P3H2, P3H3, ERO1A, PDIA4, P4HA2, GPX8 and GPX7. Moreover, based on the GSCALite, we predicted the top 10 potential drugs targeting P4HB at pan-cancer level. Future treatments for these cancers may be built on the knowledge provided by the current findings.

## Supplementary Materials

The Supplementary data can be found online at: www.aginganddisease.org/EN/10.14336/AD.2023.1126.
