# Supplementary material for: Targeting Prolyl 4-Hydroxylase Subunit Beta (P4HB) in Cancer: New Roads to Travel
Source: Aging Dis. 2023 Nov 26;15(6):2369–80. doi: 10.14336/AD.2023.1126 (PMC11567247; doi:10.14336/AD.2023.1126)
Supplement: Supplementary file 1 — The Supplementary data can be found online at: www.aginganddisease.org/EN/10.14336/AD.2023.1126. [file AD-15-6-2369-s.pdf]

SUPPLEMENTARY DATA

Targeting Prolyl 4-Hydroxylase Subunit Beta (P4HB) in Cancer: New Roads to Travel

Dechao Feng, Jie Wang, Dengxiong Li, Ruicheng Wu, Zhouting Tuo, Qingxin Yu, Luxia Ye, Akira MIYAMOTO, Koo Han Yoo, Cheng Wang, Yuanzhi Cheng, Xing Ye, Chi Zhang, Wuran Wei

© 2023. Feng D et al. Published online at http://www.aginganddisease.org/EN/10.14336/AD.2023.1126

SUPPLEMENTARY DATA

Supplementary Table 1. The correlation analysis between P4HB and immune regulatory genes at pan-cancer level.

TYPE	GENE	ACC	LUAD	LUSC	PRAD	CESC	KIRC	BLCA	KIRP	GBM	GBMLGG	LGG	UVM	KICH	KIPAN
chemokine	CXCL16	0.22895977	0.116137922	0.098661901	0.03665014	0.136445967	0.384630493	0.23161518	0.371311799	0.244843802	0.471037618	0.376164752	0.459715553	0.542355545	0.401254328
chemokine	CCL20	0.366526812	0.184207982	0.048607555	-0.007631537	0.068972322	0.19306487	0.043637774	0.183345172	0.300295287	0.387480259	0.071631043	0.240999771	0.41557511	0.392434785
chemokine	CXCL1	0.254259533	0.105165101	0.169641386	-0.038016122	0.188046319	0.221558173	0.068711177	0.264233825	0.300564593	0.228357876	0.009726522	0.193004527	0.255505547	0.293232347
chemokine	CXCL8	0.529503405	0.081972361	0.060756948	0.011988028	0.161094722	0.16905742	0.033737727	0.278340799	0.269136082	0.356163838	0.013149454	0.294662659	0.333162837	0.252367968
chemokine	CX3CL1	0.268917063	-0.01808816	0.226491198	-0.166195869	-0.044288355	-0.008447395	0.099831779	0.082741101	0.251664217	-0.289879212	-0.144835245	0.433963412	0.079469784	0.178755624
chemokine	CCL15	0.087429305	-0.065486645	-0.104401864	0.017049125	0.122437535	0.290270596	-0.103740773	-0.06475331	0.044570461	0.025310365	-0.004962987	0.043607098	0.154320547	0.322580085
chemokine	CCL28	-0.021032706	-0.071313755	0.040759099	0.009058475	0.151559939	0.107397883	0.173494081	0.165433722	0.037708278	-0.203496367	-0.30157713	-0.242065738	0.194014428	0.286975275
chemokine	CXCL17	0.099435173	0.051250795	0.07729612	-0.111822205	0.009958226	-0.150213607	0.127329746	0.200525052	0.015175279	-0.047992605	0.002836664	-0.023918804	0.034068445	-0.066652811
chemokine	CXCL2	0.279789138	0.028968463	0.070411471	-0.060067465	0.29157109	0.10134635	0.083713315	0.115827721	0.200452675	-0.001756004	-0.091488222	0.248297849	0.069105953	0.275435966
chemokine	CXCL3	0.206453274	0.011089922	0.091892062	-0.077428928	0.368682607	0.054751923	0.106483018	0.186400557	0.33893343	0.115016976	-0.104631875	0.307627153	0.281349555	0.229124783
chemokine	CXCL5	0.047177452	0.04805217	0.061288636	-0.004280094	0.112503856	0.268669046	0.153986547	0.280963217	0.296083354	-0.10430824	-0.210035707	0.152916339	0.307526307	0.367255657
chemokine	CXCL6	0.076040239	-0.048968918	0.157636442	-0.039718485	0.039373569	0.172548293	0.12319495	0.329094359	0.295061637	0.204621569	0.003778095	-0.052296059	0.209930222	0.247271746
chemokine	CCL2	0.026342079	-0.101610516	-0.035997247	-0.1397409	0.058237977	0.090903016	0.150337418	0.119411619	0.226691774	0.378393527	0.179018401	0.196885327	0.257655777	0.288765209
chemokine	CCL8	0.012084871	-0.058554205	-0.129868906	-0.199260678	0.012870467	0.106600971	0.107176506	0.170055119	0.218859842	0.269981294	0.04010603	0.225764048	0.203977246	0.248502095
chemokine	CCL13	0.114809977	-0.108343183	-0.024035857	-0.201343099	0.018522715	0.148791412	0.088385669	0.171052474	0.343575866	0.224192538	0.074372746	0.136138459	0.154684785	0.258573692
chemokine	CCL7	-0.024022933	0.040830572	-0.000589623	-0.089010689	0.048729295	0.123544523	0.196981616	0.154608864	0.293214715	0.351996509	0.069223683	0.041558683	0.301498134	0.186833277
chemokine	CCL11	0.096830216	-0.069903964	0.022640674	-0.171013371	-0.041107027	0.122636299	0.233589576	0.053939701	0.217988866	0.184986814	0.089208085	0.024670901	0.246015206	0.152141379
chemokine	CCL26	0.398251555	0.062191029	0.194195828	-0.156218419	0.005845734	0.154071056	0.145206667	0.196588978	0.300278372	0.432455049	0.25263015	0.421589922	0.343611967	0.303384021
chemokine	CCL18	0.152640837	-0.07849695	-0.054118726	0.073374595	-0.059156187	0.10115049	0.105529726	0.082270289	0.204611212	0.28898188	0.10935768	0.388948057	0.019998956	0.12317728
chemokine	CCL24	0.179177406	-0.083011915	-0.003013757	0.052725247	-0.012988461	0.098711297	0.052069236	0.145566476	0.158533091	0.193379633	0.10143502	0.416568428	0.170769789	0.158621467
chemokine	CXCL13	-0.274640902	-0.2236377	-0.241087038	-0.159937463	-0.147427878	0.173250968	0.015394248	0.210026734	0.068948618	0.178999895	0.087700499	0.41694467	0.253081329	0.319445655
chemokine	XCL1	-0.241358038	-0.099542761	-0.061432389	-0.194842392	-0.076091604	0.190429384	0.021652532	0.074687418	0.049541669	0.389539919	0.192277584	0.417380083	0.366371984	0.349450499
chemokine	XCL2	-0.373958275	-0.177413028	-0.27798177	-0.209782945	-0.067879918	0.15636095	0.015950165	0.124095792	0.05216969	0.406415617	0.265587132	0.390847876	0.218488914	0.324957003
chemokine	CXCL10	-0.145375434	-0.095188925	-0.202976783	-0.000407233	-0.175165998	0.063229692	0.104141432	0.140524012	0.015149903	0.58308597	0.367137034	0.459218782	0.237704576	0.235958103
chemokine	CXCL11	0.031329126	-0.114972663	-0.171859475	0.075336808	-0.14291203	0.037001356	0.096298825	0.132801323	-0.009341802	0.470376722	0.227058343	0.416358848	0.314354324	0.245551932
chemokine	CCL5	-0.254038778	-0.154959416	-0.162099862	-0.201675419	-0.115973191	0.160120807	0.138106415	0.211550417	0.146618116	0.515770352	0.346454009	0.561353968	0.434171798	0.35861368
chemokine	CXCL9	-0.229126955	-0.103193807	-0.253689183	-0.067618004	-0.157200877	0.10579966	0.069397638	0.146041389	0.067613911	0.541939219	0.382984313	0.48252605	0.329213812	0.308552404
chemokine	CCL1	-0.111374473	-0.056684761	-0.176486866	-0.076054282	-0.057629122	0.08106745	-0.006713143	0.078622586	-0.071216079	-0.111366312	-0.040063985	0.152667028	0.276427024	0.111551563
chemokine	CCL3	0.162001223	-0.075920727	-0.175384695	-0.122846473	-0.013165405	0.088745496	0.058313039	0.096318995	-0.123278193	-0.180418901	-0.14165044	0.5150084	0.20490982	0.241047674
chemokine	CCL4	0.102387149	-0.113367644	-0.265045142	-0.170707776	-0.024237394	0.123362905	0.055258812	0.20618009	-0.139613721	-0.136750977	-0.090602148	0.543555663	0.331621577	0.338365536
chemokine	CCL27	0.402992319	0.085341345	-0.016898336	-0.27127789	-0.135157553	-0.069891655	-0.108305894	0.067826349	0.05911365	0.364211165	0.147814536	-0.16983367	-0.068171292	0.020172538
chemokine	CCL14	-0.065066512	-0.168654722	-0.13091053	-0.043878015	-0.169190628	-0.172934345	0.019186221	-0.009521134	0.066913343	0.063871905	0.052712446	0.206780713	-0.073583133	0.008427592
chemokine	CCL16	-0.058622758	-0.137391066	-0.092073146	-0.0999101	-0.031649711	-0.229099884	-0.045842993	-0.146515069	0.041817535	0.053265957	0.057714501	0.186818093	-0.152353363	-0.00537197
chemokine	CCL19	-0.069762807	-0.225353562	-0.195855998	-0.10406066	-0.180282848	0.127485526	0.003426794	0.161721337	-0.172507047	-0.284419293	-0.109763374	0.185861044	0.330013754	0.267221142
chemokine	CCL21	0.018339951	-0.043479409	-0.111235969	-0.131355903	-0.200717081	0.002091674	0.080311168	0.149420455	-0.165013064	-0.136056596	0.02579952	0.184520469	0.043273145	-0.007744394
chemokine	CXCL12	-0.082103727	-0.188008381	-0.089570529	-0.234126704	-0.044457671	0.102885673	0.142699899	0.179184914	0.171832947	-0.021014393	0.037442958	0.400167981	0.394180087	0.317651261
chemokine	CCL17	-0.119252389	-0.110689375	-0.060553919	-0.098267071	-0.166288595	0.081916672	-0.034899841	0.055527967	-0.008143271	0.143470787	0.09805249	0.415692864	0.125412742	0.227727914
chemokine	CCL22	0.311193007	0.033698434	-0.051350784	-0.065306822	-0.075127451	0.109978989	0.023336047	0.098764397	-0.070794079	0.146949438	0.136880003	0.415502297	0.163762333	0.250803196
chemokine	CCL23	0.014918329	-0.157479737	-0.097453235	-0.011268687	-0.059439669	0.055383054	0.079838564	0.106197392	-0.030341365	0.36807195	0.216949462	0.437944171	0.162236701	0.233954218
chemokine	CCL25	0.12996735	-0.10093265	-0.166551201	-0.004254304	0.000570069	0.070543274	-0.007488592	0.040631889	0.006965675	0.196787718	0.169503721	0.073084655	0.022445193	0.002745576
chemokine	CXCL14	0.275283059	-0.018597339	0.015460882	-0.050799397	-0.079676137	0.241428149	-0.078536474	0.160163747	0.259990317	0.182413798	-0.030469861	0.102312453	0.314629599	0.383922078
receptor	CCR9	-0.275670415	-0.188112671	0.016486633	-0.040982989	0.056522118	0.107378052	-0.031980903	0.052808023	-0.063205755	-0.160486414	-0.168579843	0.20900793	-0.033049275	0.014310018
receptor	CX3CR1	-0.382165024	-0.059368711	-0.120314691	-0.097810754	-0.096110797	0.135424281	0.021397883	0.030718892	-0.114757435	0.000792726	-0.00078854	0.047734122	0.256359405	0.257870679
receptor	CXCR5	0.401130463	0.177774523	0.057737255	-0.133482167	-0.043671007	0.100902166	0.180166243	0.154511025	0.106966229	0.142602276	0.146005293	0.201375706	0.079117165	0.183935624
receptor	CXCR1	0.202220812	-0.003966291	0.006905276	0.043980742	0.076700724	0.028763451	0.028135605	0.245503201	0.176205317	0.163796858	-0.0431353	0.14916109	0.315657862	0.238762957
receptor	CXCR2	0.185230441	-0.05607819	-0.063955062	-0.051806053	-0.05091861	0.07317087	0.040740293	0.269910039	0.086997278	0.395320786	0.222959823	0.253791304	0.32776488	0.275123043
receptor	CCR1	-0.054875925	-0.086114923	-0.080545571	-0.153355196	-0.079726228	0.253930736	0.245865836	0.241996387	0.235442728	0.36513129	0.221964804	0.384068482	0.362059248	0.400291736
receptor	CCR3	-0.225492962	-0.000185425	-0.033081636	-0.170459566	-0.085989523	0.042845082	0.278086788	0.164132166	0.144432573	0.292237424	0.121337147	0.210885427	0.352569853	0.163789792
receptor	CCR8	-0.058312128	-0.07567141	-0.113901397	-0.089923174	-0.110349664	0.245272725	0.068605802	0.104657151	0.233181464	0.324514871	0.225948667	0.236735926	0.169562389	0.326889746
receptor	CCR5	-0.199245046	-0.130680655	-0.172573133	-0.154969744	-0.089607129	0.195928545	0.096008551	0.210657392	0.253374145	0.505536327	0.337296482	0.394392051	0.531171398	0.396341578
receptor	CXCR6	-0.276228449	-0.184939576	-0.253310223	-0.117454849	-0.048444826	0.223591972	0.081802322	0.174317178	0.044346877	0.464768623	0.341241871	0.459472325	0.514433688	0.383307032
receptor	CXCR3	-0.179723774	-0.10234513	-0.227375592	-0.14541795	-0.090210262	0.199569764	0.03073877	0.135046276	0.16667896	0.508546606	0.392840451	0.502571992	0.452277816	0.372661342
receptor	CCR2	-0.285355563	-0.213989477	-0.188765082	-0.115722737	-0.110399004	0.170391183	0.093745937	0.180225675	0.162510682	0.447630425	0.26324175	0.232622458	0.481814538	0.374480666
receptor	CCR4	-0.053372154	-0.218755917	-0.197475422	-0.114470813	-0.084951007	0.14001954	0.034453961	0.204203781	0.187088814	0.443066464	0.248506043	0.252868111	0.446943455	0.348808071

© 2023. Feng D et al. Published online at http://www.aginganddisease.org/EN/10.14336/AD.2023.1126

SUPPLEMENTARY DATA

TYPE	GENE	ACC	LUAD	LUSC	PRAD	CESC	KIRC	BLCA	KIRP	GBM	GBMLGG	LGG	UVM	KICH	KIPAN
receptor	CCR6	-0.162888538	-0.155894445	-0.132589843	-0.071511585	-0.094699953	0.232188343	-0.043744637	0.213335582	0.252986217	0.20776093	0.0271832	0.338476119	0.26627254	0.386399601
receptor	CCR7	-0.069566544	-0.143824738	-0.149472391	-0.120876437	-0.134686967	0.210897634	0.009635942	0.275810572	0.196203298	0.455585203	0.295929742	0.339491779	0.388216995	0.383022113
receptor	XCR1	-0.18252393	-0.159149481	-0.100046524	-0.042180786	-0.070598752	0.118989456	0.250983146	0.193434937	0.213039175	0.363530714	0.255597867	0.348810744	0.281915477	0.319471682
receptor	CCR10	0.253623815	0.027052372	0.003056149	-0.122089625	0.053824971	-0.046228216	0.147216613	0.322920056	0.194604719	0.537749632	0.401013937	0.406505456	0.117311734	0.250706267
receptor	CXCR4	0.278694989	-0.165582782	-0.186477408	-0.233802763	-0.081292854	0.164538825	0.062485385	0.236963305	0.290633487	0.547517457	0.34654985	0.493482151	0.517085899	0.386317569
MHC	HLA-DMB	0.347836374	-0.177275846	-0.24336538	0.049530098	0.065744204	0.152072983	0.146070818	0.156792224	-0.113150713	0.503062217	0.359360697	0.447312849	0.271373253	0.299350867
MHC	HLA-DQB1	-0.079591984	0.004483917	-0.114047926	-0.133821563	0.021106884	0.12354218	0.03715568	0.115918994	0.115548184	0.417225732	0.257223278	0.474771633	0.18638973	0.290224286
MHC	HLA-DMA	0.05836269	-0.072283502	-0.157413691	-0.143823894	0.128301141	0.190199751	0.153010121	0.22273292	0.09757875	0.511337841	0.389490761	0.564800389	0.262710184	0.386552023
MHC	HLA-DOA	-0.158979921	-0.177233876	-0.159834325	-0.18234634	0.016859296	0.177500977	0.109575401	0.219621482	0.122230487	0.488763424	0.363050293	0.517104759	0.345336506	0.364507472
MHC	HLA-DPB1	-0.074451864	-0.160615454	-0.190490216	-0.126604268	0.005833811	0.222501662	0.144185371	0.167137413	0.130992808	0.541109918	0.42017522	0.483471276	0.378043816	0.381966412
MHC	HLA-DPA1	-0.116383616	-0.145762206	-0.20349273	-0.104901104	-0.015776666	0.204538165	0.137134605	0.125676701	0.060458785	0.530178137	0.37722261	0.556478557	0.310051143	0.355635758
MHC	HLA-DRA	-0.078921079	-0.174549369	-0.22948412	-0.147876788	0.050036306	0.227410914	0.128059913	0.121336606	0.028332935	0.542922349	0.387048486	0.501168452	0.324997391	0.37486798
MHC	HLA-DQA1	-0.193900756	-0.060774205	-0.190164894	-0.140793691	-0.014225734	0.205924388	0.098205909	0.140828999	0.083901475	0.501559481	0.371765011	0.533839666	0.361862019	0.356655728
MHC	HLA-DRB1	-0.125559477	-0.068596305	-0.175075845	-0.119159446	0.070130248	0.222766414	0.139969354	0.150162901	0.14567191	0.548071569	0.388618401	0.589049349	0.262164701	0.388777706
MHC	HLA-DOB	-0.053295992	-0.19017086	-0.196008682	-0.222580123	0.069008805	0.081548122	0.147157037	0.162738946	0.032506078	0.363442759	0.237697513	0.477417036	0.366006325	0.321276896
MHC	HLA-DQA2	-0.176306232	-0.099463471	-0.137939421	-0.058272605	-0.094716165	0.137932481	0.102430543	0.115136683	0.092472418	0.394201012	0.295968769	0.318229965	0.21347306	0.264673068
MHC	HLA-F	-0.001367054	-0.111568664	-0.177792337	-0.076442302	0.04661859	0.019386312	0.001270908	0.204810885	0.127280525	0.485715826	0.431499345	0.445009737	0.136791567	0.303422849
MHC	TAPBP	0.380198749	0.172863523	0.130276937	0.157395764	0.191248512	0.255108167	0.217411174	0.338761803	0.475854934	0.579953033	0.627868509	0.523968622	0.65924225	0.396265145
MHC	TAP1	0.533045901	-0.01041738	-0.152270901	0.132128048	0.026861244	0.169047007	0.122346932	0.348213958	0.239952086	0.633617743	0.564052314	0.587049659	0.541243516	0.416981989
MHC	TAP2	0.542273516	0.068929821	0.037995773	0.158030648	0.004412519	0.085887766	0.162295142	0.382829188	0.334690342	0.487320715	0.554527431	0.435022335	0.330424799	0.355274567
MHC	HLA-G	0.268281944	0.015097486	-0.093546834	0.091679365	0.080614666	0.116503028	0.045094834	0.189442944	0.17949006	0.324807564	0.20446733	0.201029736	0.204876025	0.304559402
MHC	HLA-E	0.052184657	-0.083194781	-0.042362478	-0.07068484	0.008145254	-0.00724251	0.139565299	0.351097422	0.197789256	0.404081543	0.302251689	0.546032132	0.340611627	0.321207588
MHC	B2M	0.175692728	-0.108858948	-0.198533932	0.150488872	0.095281236	0.008348816	0.123954519	0.208574541	0.040140559	0.562577691	0.437526001	0.501119766	0.282872352	0.181258669
MHC	HLA-C	0.163915032	-0.008683401	-0.15542479	0.290044033	0.200970259	0.14402565	0.243332871	0.231694121	0.206033649	0.568542235	0.445319957	0.467502434	0.249932157	0.279962197
MHC	HLA-A	-0.01840265	0.060282803	-0.078927362	0.079187944	0.176201996	0.209692082	0.082949226	0.244309468	0.323287033	0.675162673	0.547156675	0.606621227	0.352468427	0.371561105
MHC	HLA-B	0.070008938	-0.062779955	-0.144122223	0.135058602	0.12006193	0.105201044	0.102864632	0.258225091	0.257715456	0.642653028	0.504720487	0.481450828	0.335184219	0.327940945
Immunoinhibitor	IL10RB	0.689731321	-0.01654031	0.182693981	0.271746897	0.218806182	0.376853664	0.321987779	0.436045025	0.424432066	0.750985009	0.637957923	0.623226772	0.439265212	0.531551974
Immunoinhibitor	PVRL2	0.398916873	0.373443651	0.336611165	0.577736802	0.288555771	0.159069482	0.462889374	0.4750814	0.574783758	0.701203766	0.535402768	0.469233625	0.265820539	0.179589558
Immunoinhibitor	ADORA2A	0.299832397	-0.123433642	-0.092922381	-0.205592307	0.093285361	0.004534294	0.087053073	0.204320249	0.163941553	0.085165368	0.186225373	0.425595366	0.299584577	0.281377634
Immunoinhibitor	BTLA	0.033148683	-0.25456589	-0.24155266	-0.108725614	-0.133629272	0.052196095	0.0425313	0.193151152	-0.04083364	0.183324629	0.099323531	0.339688381	0.33962156	0.239598039
Immunoinhibitor	CD274	-0.039915857	-0.041388963	-0.017907629	-0.188715845	-0.040806136	-0.078382176	0.122700732	0.012307865	0.383669979	0.385269561	0.152820841	0.214845653	-0.010521701	-0.051998663
Immunoinhibitor	IDO1	0.319844363	-0.071014074	-0.112588334	-0.062585733	-0.002002837	0.02046244	0.026029237	0.161747677	0.151528987	0.569532705	0.411748381	0.484352264	0.138653752	0.248084946
Immunoinhibitor	CD160	-0.009021213	-0.200326861	-0.202126723	-0.162196559	0.017848908	-0.022103955	0.006658573	0.123144964	0.088480341	0.188612085	0.21349725	0.209884183	0.268856515	0.199920438
Immunoinhibitor	KIR2DL1	-0.196146807	-0.04710085	-0.083592942	-0.045370857	-0.020321645	0.066918305	0.037084035	0.099731209	0.056620747	0.219057881	0.159480563	0.222077176	0.129684529	0.269451862
Immunoinhibitor	KIR2DL3	-0.364565832	-0.005101371	-0.105373253	-0.006684672	0.006034449	0.098865829	0.025328173	0.102809445	0.120048291	0.259645701	0.231845349	0.327149472	0.336520998	0.259890444
Immunoinhibitor	LAG3	-0.055962081	-0.053334132	-0.185212166	-0.301210496	-0.025056262	0.159680371	0.073979199	0.275566482	0.092824138	0.337675116	0.32787043	0.554624846	0.402643811	0.390220255
Immunoinhibitor	CTLA4	-0.224385134	-0.123863498	-0.22489154	-0.208966911	-0.127474141	0.10574654	0.031552179	0.145981201	0.155555022	0.263454489	0.114063401	0.31715621	0.45774624	0.31844288
Immunoinhibitor	TIGIT	-0.267317077	-0.130202261	-0.212435062	-0.167618435	-0.092336025	0.144196806	0.02462879	0.238207816	0.118269817	0.09680015	0.111493618	0.412311049	0.372634672	0.350825073
Immunoinhibitor	CD96	-0.124151259	-0.23797449	-0.294997215	-0.19889107	-0.113650382	0.145973472	-0.025221877	0.199167459	0.085267221	0.467065411	0.321459055	0.489232878	0.431812263	0.361092967
Immunoinhibitor	PDCD1	-0.148511811	-0.055929314	-0.240598651	-0.177359657	-0.042752994	0.167634849	0.051162444	0.239894152	0.171777913	0.501803787	0.399373777	0.519645982	0.381763726	0.365649471
Immunoinhibitor	CD244	-0.19177307	-0.188800609	-0.218186468	-0.088024269	-0.043535854	0.095601055	0.150172346	0.203185745	-0.025749919	0.198411306	-2.61E-05	0.21667501	0.347155109	0.334255813
Immunoinhibitor	PDCD1LG2	-0.097707242	-0.164659591	-0.109515625	-0.219792554	-0.037749018	0.100931362	0.163825435	0.22302436	0.107494258	0.484450327	0.281217118	0.115627422	0.391636138	0.324319899
Immunoinhibitor	VTCN1	0.114145859	0.063332725	0.203892089	-0.149109148	0.217555559	-0.008034275	0.294609293	0.108760948	0.011703547	-0.111923014	0.010512495	0.10220192	0.419517311	0.000693055
Immunoinhibitor	KDR	-0.065120375	0.10174469	0.049527231	-0.038168464	0.187865901	-0.026587677	0.084340765	0.035546712	0.347015738	0.284278634	0.168470219	0.550187141	0.023567477	0.177054466
Immunoinhibitor	TGFBR1	0.511294701	0.011414691	0.079381349	-0.07982641	0.078775854	0.082589377	0.170016621	0.26153008	0.144814513	0.479505145	0.411908327	-0.043050932	0.247594197	0.206441618
Immunoinhibitor	TGFB1	0.028865871	0.043965244	0.237324949	-0.207950598	-0.080052418	0.273440105	0.041169414	0.325902393	0.430038767	0.521138132	0.379564838	0.532789679	0.177876587	0.454505994
Immunoinhibitor	LGALS9	0.058310111	0.009207826	-0.116668165	-0.217905298	0.130552115	0.290105487	0.132576997	0.336329496	0.071512457	0.411694797	0.351665349	0.526825706	0.38986306	0.464527107
Immunoinhibitor	CSF1R	-0.184184237	-0.037891064	-0.078535185	-0.174011873	-0.120594365	0.272523739	0.156399146	0.222765462	0.206613308	0.090048833	0.06831237	0.381413748	0.446889123	0.416089618
Immunoinhibitor	HAVCR2	-0.002550182	-0.103869133	-0.189243166	-0.199741236	-0.104019447	0.121993706	0.143724975	-0.002424862	-0.016965934	0.398564637	0.285618813	0.479184928	0.3286433	0.279334341
Immunoinhibitor	IL10	0.080124232	-0.168247803	-0.129224296	-0.141845409	0.038047716	0.195141173	0.177231331	0.206436695	0.034430533	0.380511847	0.185595349	0.487746213	0.368799543	0.349876129
Immunostimulator	CD276	0.497624071	0.350021321	0.362435387	0.213268639	0.21507969	0.354949797	0.373525074	0.475090424	0.696584451	0.793176197	0.654985624	0.711002921	0.470101974	0.444252257
Immunostimulator	PVR	0.178468617	0.296821669	0.388621674	0.304684626	0.223080053	0.284416016	0.293447797	0.477531582	0.597056458	0.391858221	0.154776253	0.569870617	0.119256863	0.177033088
Immunostimulator	CXCL12	-0.082103727	-0.188008381	-0.089570529	-0.234126704	-0.044457671	0.102885673	0.142699899	0.179184914	0.171832947	-0.021014393	0.037442958	0.400167981	0.394180087	0.317651261
Immunostimulator	TNFRSF8	0.218883874	-0.07967299	-0.091479483	-0.145486455	-0.07102707	0.181894713	0.145181892	0.235235202	0.279092215	0.191459373	0.122002177	0.60791351	0.30093175	0.370721398
Immunostimulator	TNFSF4	0.281607194	-0.063478097	-0.094830579	-0.022447515	-0.020360943	0.125594651	0.167497859	0.19427509	0.408189132	0.277791599	0.10632094	0.148432824	0.440785582	0.323806678
Immunostimulator	ULBP1	0.254072703	0.023725546	0.200187715	0.02655439	0.052962183	0.142237701	0.27512284	0.22334583	0.212816908	0.443389762	0.220291097	0.444795961	0.20686634	0.217811576
Immunostimulator	NT5E	0.209505156	-0.004795976	0.125111248	-0.148291654	0.251877535	0.079625277	0.042807786	0.211995939	0.337098887	0.228305954	0.164764297	0.40829119	0.397010626	0.291712692

© 2023. Feng D et al. Published online at http://www.aginganddisease.org/EN/10.14336/AD.2023.1126

SUPPLEMENTARY DATA

Immunostimulator	TNFSF15	0.093205593	0.004490403	0.044790552	-0.019281262	0.185871997	-0.116189047	0.152733652	0.041051355	0.478320184	0.35174661	0.100270481	0.404743556	0.269124386	-0.131019857
**TYPE**	**GENE**	**ACC**	**LUAD**	**LUSC**	**PRAD**	**CESC**	**KIRC**	**BLCA**	**KIRP**	**GBM**	**GBMLGG**	**LGG**	**UVM**	**KICH**	**KIPAN**
Immunostimulator	ICOSLG	0.066612329	0.072673375	0.085544527	-0.039433509	-0.078104334	0.11999756	0.220031357	-0.000619908	0.212989955	0.205945543	0.129650595	0.619855637	0.279403783	0.198370226
Immunostimulator	RAET1E	0.415998424	0.089702801	0.017903443	-0.049975406	-0.062765253	-0.012801393	0.105578547	0.110706868	0.036112089	0.038564394	0.038881371	0.009168161	0.225821955	0.218647859
Immunostimulator	CD40	-0.19278217	-0.081386172	0.045882004	-0.206323196	0.048092507	0.193570466	-0.063507064	0.294501379	0.285368689	0.460149175	0.221057582	-0.227934688	0.190777388	0.40690678
Immunostimulator	ENTPD1	0.177902216	-0.162266605	-0.153135854	-0.180355315	-0.023637317	0.040907228	0.117060801	0.298559245	0.111392006	0.423291425	0.24962803	-0.138559753	0.338353949	0.333334748
Immunostimulator	TNFRSF14	0.163930332	-0.058452308	-0.11965326	0.152852924	0.069470317	-0.068540442	-0.058394963	-0.109891816	0.310996296	0.537516634	0.299180904	0.51630964	0.089072122	0.092831564
Immunostimulator	IL6R	0.045915517	-0.005202386	0.14897129	0.005021528	-0.03734322	0.099138408	-0.013505137	0.194902759	0.225342627	0.154449774	0.148620523	0.264771932	0.535654643	0.329702551
Immunostimulator	TNFSF14	-0.117226767	-0.019021264	-0.05052179	-0.156260043	0.084329757	0.275307292	-0.019647893	0.274375841	0.265735948	0.532641952	0.345722447	0.37343427	0.373340354	0.44286439
Immunostimulator	C10orf54	0.070509366	-0.150882028	-0.087044533	-0.162996414	-0.001737586	0.174285087	0.120159351	0.246957276	-0.0681066	-0.12331761	-0.021712252	0.557684935	0.42402672	0.410044962
Immunostimulator	TNFSF9	0.143613001	0.081645559	0.036639081	-0.118565024	-0.037078889	0.30263851	0.024439273	0.257847037	-0.045121361	-0.189869356	-0.195229974	0.633559727	0.204662027	0.408921528
Immunostimulator	TMEM173	0.040302333	0.0137313	0.131539129	-0.188707961	-0.01199738	0.202557904	0.114367677	0.372102656	0.386855475	0.499038426	0.326105112	0.634810127	0.381484187	0.373801127
Immunostimulator	TNFSF13	-0.010095234	0.045093086	-0.070521155	0.207611212	0.239740925	0.131479045	0.192086169	0.189965572	0.215827724	0.34223209	0.163178488	0.344230769	0.213109279	0.106614512
Immunostimulator	CD28	0.077532875	-0.195402545	-0.15493526	-0.123416213	-0.177184112	0.178046122	0.038135493	0.22250924	0.135306185	0.411111251	0.208075554	0.473430799	0.358225011	0.35680672
Immunostimulator	ICOS	-0.221031528	-0.162854362	-0.222313846	-0.169253461	-0.129146928	0.133895721	0.000499363	0.172057085	0.054344229	0.444193671	0.316539306	0.346481683	0.358965944	0.347941343
Immunostimulator	LTA	0.065917481	-0.134914807	-0.200534817	-0.155621024	-0.135539492	0.181355951	0.046163669	0.219540353	0.006904075	0.133824908	0.150389986	0.429415442	0.387422699	0.376907565
Immunostimulator	CD27	-0.265592959	-0.19450973	-0.226084459	-0.186700302	-0.120647615	0.152758614	0.005426035	0.172842843	0.132096161	0.424050185	0.422851267	0.449326875	0.449450404	0.361401324
Immunostimulator	CD40LG	-0.160074671	-0.209364985	-0.225146155	-0.14049242	-0.105826818	0.154985791	-0.048179751	0.151912033	-0.01501678	0.368715406	0.273860767	0.379176501	0.480692889	0.355792111
Immunostimulator	CD48	-0.234787584	-0.28856694	-0.297184714	-0.193361309	-0.160654338	0.113295673	0.017444627	0.158825455	-0.103782704	0.569114393	0.419439216	0.276312426	0.428173307	0.339213622
Immunostimulator	CD70	-0.017597873	-0.194327104	-0.183830191	-0.144183875	-0.15107749	0.300094147	-0.021707235	0.108007264	0.205994289	0.538774914	0.31911009	0.487013703	0.263591534	0.437923162
Immunostimulator	TNFRSF18	-0.170043479	0.082986157	0.143173491	-0.169601067	-0.038644656	0.190272747	0.003604667	0.227241341	0.350920358	0.316727539	0.072484025	0.50930492	0.262350197	0.323607334
Immunostimulator	IL2RA	0.084336766	-0.042452366	-0.120664298	-0.170914633	-0.064456136	0.199576716	0.071146114	0.353203001	0.328171401	0.520523844	0.292630345	0.32946643	0.401465979	0.377598123
Immunostimulator	CD80	0.237714771	-0.185676796	-0.179306501	-0.205969524	-0.130210651	0.151785721	0.08388825	0.266349899	0.074597155	0.5301421	0.352737516	0.44102873	0.34573172	0.323794121
Immunostimulator	CD86	-0.074323836	-0.130268651	-0.198706454	-0.232407103	-0.184718561	0.253970093	0.116312281	0.217497482	-0.015985901	0.329439121	0.210984209	0.449449152	0.43155961	0.400031891
Immunostimulator	TNFRSF9	0.181619723	-0.061719331	-0.15108479	-0.135402428	-0.049687181	0.232026882	0.100806695	0.269245333	0.168843281	0.421062583	0.297671227	0.480608305	0.373150675	0.423892581
Immunostimulator	IL6	0.158124497	-0.042730985	0.072914925	-0.07332483	0.166012234	0.217920638	0.089579024	0.251005682	0.263842491	0.39197393	0.190442686	0.337264355	0.071139975	0.336825172
Immunostimulator	TNFRSF4	0.13478981	0.034042959	-0.090929356	-0.189434418	-0.0794361	-0.075158575	0.069890571	0.233153664	0.178744589	0.576375955	0.381830879	0.466325029	0.110452373	0.231684812
Immunostimulator	TNFSF13B	0.492513967	-0.187561534	-0.21743804	-0.208911279	-0.122785752	0.237372297	0.071685896	0.262894183	0.052317146	0.42309442	0.331615406	0.368569072	0.483392833	0.418458918
Immunostimulator	CXCR4	0.278694989	-0.165582782	-0.186477408	-0.233802763	-0.081292854	0.164538825	0.062485385	0.236963305	0.290633487	0.547517457	0.34654985	0.493482151	0.517085899	0.386317569
Immunostimulator	MICB	0.16088494	-0.009642726	0.012288016	-0.163427983	0.129467958	0.126443998	0.274311037	0.275104946	0.18438417	0.514666982	0.323125131	0.411483663	0.562779245	0.339842191
Immunostimulator	TMIGD2	-0.092985808	-0.079300977	-0.195261314	-0.083073239	-0.040421722	0.182302301	0.023437948	0.210824968	-0.316320829	-0.213688033	-0.04109346	-0.144882712	0.293731905	0.293523127
Immunostimulator	TNFRSF25	0.089007782	0.104136957	-0.106831315	-0.273094631	-0.100311329	-0.047043166	-0.048836733	0.044208708	0.182166802	0.091371235	-0.006188563	0.243347215	0.224537305	0.188821973
Immunostimulator	TNFRSF13B	-0.117940333	-0.191701523	-0.16607995	-0.162923983	-0.18600525	0.100507164	-0.016706265	0.116019182	0.028732557	-0.070023255	-0.069573742	0.179466431	0.22386768	0.251159692
Immunostimulator	TNFRSF17	-0.024634426	-0.235099497	-0.205508347	-0.136138391	-0.008052288	0.071847123	0.005596631	0.144429781	-0.051443844	0.061264387	0.068816261	0.222605231	0.190979108	0.258444077
Immunostimulator	TNFSF18	-0.085527449	-0.183373875	-0.100768282	-0.129357585	-0.10357827	0.048285051	0.008061697	0.224152031	-0.196434078	-0.081162489	-0.092392621	0.330607886	0.242691751	0.278890553
Immunostimulator	KLRC1	0.058388921	-0.156335351	-0.255776178	-0.148840072	-0.001578318	0.067251046	0.057351103	0.119756022	-0.015447406	-0.012843514	0.091952989	0.409587385	0.265938487	0.219321222
Immunostimulator	KLRK1	0.005626656	-0.212840554	-0.269375606	-0.203971023	-0.050544072	-0.00096348	0.042670841	0.189235553	-0.020905115	-0.253207235	0.006742848	0.421415949	0.371972614	0.294168005
Immunostimulator	HHLA2	0.129960556	-0.041587892	0.05585738	-0.01090576	0.095099718	0.102946447	-0.00835652	-0.005043395	-0.127679952	-0.379661086	-0.294817754	-0.059373699	0.321847492	0.284776843
Immunostimulator	BTNL2	-0.18153016	-0.141058551	-0.020159075	-0.013087547	-0.060967701	-0.115225833	-0.012373548	0.032664136	-0.096564882	0.013614045	-0.064287896	-0.165303866	-0.121977899	-0.000828865
Immunostimulator	TNFRSF13C	0.464617797	-0.203425384	-0.022836434	-0.245948541	-0.09577618	-0.123317269	-0.090278471	0.206263511	-0.14149969	-0.31699946	-0.155654251	-0.18035961	0.171092074	0.014501616
		**0 < absolute value of r < 0.3**					**0.3 < absolute value of r < 0.5**			**absolute value of r > 0.5**				**p > 0.05**	

© 2023. Feng D et al. Published online at http://www.aginganddisease.org/EN/10.14336/AD.2023.1126

SUPPLEMENTARY DATA

Supplementary Table 2. The correlation analysis between P4HB and immune checkpoints at pan-cancer level.

TYPE	GENE	LUAD	LUSC	CESC	PRAD	ACC	GBMLGG	LGG	UVM	KICH	KIPAN	GBM	KIRC	KIRP	BLCA
Inhibitory	CD276	0.350021321	0.362435387	0.21507969	0.213268639	0.497624071	0.793176197	0.654985624	0.711002921	0.470101974	0.444252257	0.696584451	0.354949797	0.475090424	0.373525074
Inhibitory	VEGFA	0.284209315	0.275423914	0.218603936	0.089699773	0.256086019	0.605437075	0.352053613	0.27966504	0.19586682	0.201304394	0.4269508	-0.080516215	0.050157243	0.027252642
Inhibitory	CD274	-0.041388963	-0.017907629	-0.040806136	-0.188715845	-0.039915857	0.385269561	0.152820841	0.214845653	-0.010521701	-0.051998663	0.383669979	-0.078382176	0.012307865	0.122700732
Inhibitory	KIR2DL1	-0.04710085	-0.083592942	-0.020321645	-0.045370857	-0.196146807	0.219057881	0.159480563	0.222077176	0.129684529	0.269451862	0.056620747	0.066918305	0.099731209	0.037084035
Inhibitory	KIR2DL3	-0.005101371	-0.105373253	0.006034449	-0.006684672	-0.364565832	0.259645701	0.231845349	0.327149472	0.336520998	0.259890444	0.120048291	0.098865829	0.102809445	0.025328173
Inhibitory	HAVCR2	-0.103869133	-0.189243166	-0.104019447	-0.199741236	-0.002550182	0.398564637	0.285618813	0.479184928	0.3286433	0.279334341	-0.016965934	0.121993706	-0.002424862	0.143724975
Inhibitory	IL10	-0.168247803	-0.129224296	0.038047716	-0.141845409	0.080124232	0.380511847	0.185595349	0.487746213	0.368799543	0.349876129	0.034430533	0.195141173	0.206436695	0.177231331
Inhibitory	IDO1	-0.071014074	-0.112588334	-0.002002837	-0.062585733	0.319844363	0.569532705	0.411748381	0.484352264	0.138653752	0.248084946	0.151528987	0.02046244	0.161747677	0.026029237
Inhibitory	CTLA4	-0.123863498	-0.22489154	-0.127474141	-0.208966911	-0.224385134	0.263454489	0.114063401	0.31715621	0.45774624	0.31844288	0.155555022	0.10574654	0.145981201	0.031552179
Inhibitory	TIGIT	-0.130202261	-0.212435062	-0.092336025	-0.167618435	-0.267317077	0.09680015	0.111493618	0.412311049	0.372634672	0.350825073	0.118269817	0.144196806	0.238207816	0.02462879
Inhibitory	PDCD1	-0.055929314	-0.240598651	-0.042752994	-0.177359657	-0.148511811	0.501803787	0.399373777	0.519645982	0.381763726	0.365649471	0.171777913	0.167634849	0.239894152	0.051162444
Inhibitory	LAG3	-0.053334132	-0.185212166	-0.025056262	-0.301210496	-0.055962081	0.337675116	0.32787043	0.554624846	0.402643811	0.390220255	0.092824138	0.159680371	0.275566482	0.073979199
Inhibitory	SLAMF7	-0.161981083	-0.242293803	-0.105351879	-0.157735386	-0.135895656	0.497545698	0.371707803	0.295673239	0.356057999	0.329728398	-0.039847399	0.125858588	0.227874901	0.043166425
Inhibitory	ADORA2A	-0.123433642	-0.092922381	0.093285361	-0.205592307	0.299832397	0.085165368	0.186225373	0.425595366	0.299584577	0.281377634	0.163941553	0.004534294	0.204320249	0.087053073
Inhibitory	BTLA	-0.25456589	-0.24155266	-0.133629272	-0.108725614	0.033148683	0.183324629	0.099323531	0.339688381	0.33962156	0.239598039	-0.04083364	0.052196095	0.193151152	0.0425313
Inhibitory	IL13	-0.14699837	-0.130332933	-0.042714931	-0.112484574	0.148525565	0.044998744	0.134426842	0.063853894	0.227290096	0.083388948	0.011719075	-0.066285115	0.054429031	-0.080151275
Inhibitory	IL4	-0.031078731	-0.061360207	-0.10216155	-0.118432389	0.109641636	0.411059277	0.284053473	-0.143608224	0.016277207	0.004755745	0.160091918	-0.117008102	0.071635756	-0.015823595
Inhibitory	ARG1	-0.188430903	0.001951203	-0.054238018	-0.052870121	0.302989297	0.092000399	-0.129260924	-0.189937956	0.10780902	0.086637663	0.084796464	-0.111270352	0.063935548	-0.11456089
Inhibitory	EDNRB	-0.119646629	-0.086820825	-0.01756573	-0.092822088	0.406375288	-0.256495452	-0.142070602	-0.383617332	-0.080555266	0.129753541	-0.129341057	-0.146157107	0.087060214	0.07252694
Inhibitory	IL12A	-0.064134495	-0.031804985	0.102114271	-0.195110941	0.098870343	-0.125790805	-0.070724559	-0.012177955	0.154898106	0.189388916	0.021199262	-0.040606841	0.227229684	-0.048027167
Inhibitory	TGFB1	0.043965244	0.237324949	-0.080052418	-0.207950598	0.028865871	0.521138132	0.379564838	0.532789679	0.177876587	0.454505994	0.430038767	0.273440105	0.325902393	0.041169414
Inhibitory	C10orf54	-0.150882028	-0.087044533	-0.001737586	-0.162996414	0.070509366	-0.12331761	-0.021712252	0.557684935	0.42402672	0.410044962	-0.0681066	0.174285087	0.246957276	0.120159351
Inhibitory	VEGFB	0.31232394	0.193758263	0.091976735	0.040207234	0.51104159	0.34418868	0.341460531	0.137682571	0.351174199	0.05300692	0.193567788	0.262375347	0.101492429	0.296172229
Inhibitory	VTCN1	0.063332725	0.203892089	0.217555559	-0.149109148	0.114145859	-0.111923014	0.010512495	0.10220192	0.419517311	0.000693055	0.011703547	-0.008034275	0.108760948	0.294609293
Stimulaotry	CD27	-0.19450973	-0.226084459	-0.120647615	-0.186700302	-0.265592959	0.424050185	0.422851267	0.449326875	0.449450404	0.361401324	0.132096161	0.152758614	0.172842843	0.005426035
Stimulaotry	CD28	-0.195402545	-0.15493526	-0.177184112	-0.123416213	0.077532875	0.411111251	0.208075554	0.473430799	0.358225011	0.35680672	0.135306185	0.178046122	0.22250924	0.038135493
Stimulaotry	CD40LG	-0.209364985	-0.225146155	-0.105826818	-0.14049242	-0.160074671	0.368715406	0.273860767	0.379176501	0.480692889	0.355792111	-0.01501678	0.154985791	0.151912033	-0.048179751
Stimulaotry	ICOS	-0.162854362	-0.222313846	-0.129146928	-0.169253461	-0.221031528	0.444193671	0.316539306	0.346481683	0.358965944	0.347941343	0.054344229	0.133895721	0.172057085	0.000499363
Stimulaotry	SELP	-0.117268522	-0.087636075	-0.074666341	-0.048731608	-0.245364861	0.106310513	0.026165966	0.203324633	0.119012578	0.208750173	0.160619612	0.001697862	0.102336552	-0.045351847
Stimulaotry	IFNG	-0.12165923	-0.237299011	-0.042942125	-0.167528034	-0.069618645	0.234498223	0.195988495	0.436285088	0.206943788	0.330993627	0.013787351	0.133746106	0.136475145	0.021342405
Stimulaotry	IL2	-0.133487423	-0.195163805	-0.030591374	-0.099867482	-0.094506921	0.179316421	0.148970811	0.258431077	0.190362368	0.220217639	0.068300644	0.065288529	0.046907062	-0.071241902
Stimulaotry	TNF	-0.076852847	0.028701666	0.104946433	-0.100789918	-0.013345888	-0.106890629	-0.084454979	0.468054747	0.360563395	0.192150794	-0.11759591	0.058881812	0.144642001	0.119961936
Stimulaotry	TNFSF9	0.081645559	0.036639081	-0.037078889	-0.118565024	0.143613001	-0.189869356	-0.195229974	0.633559727	0.204662027	0.408921528	-0.045121361	0.30263851	0.257847037	0.024439273
Stimulaotry	ICOSLG	0.072673375	0.085544527	-0.078104334	-0.039433509	0.066612329	0.205945543	0.129650595	0.619855637	0.279403783	0.198370226	0.212989955	0.11999756	-0.000619908	0.220031357
Stimulaotry	TNFSF4	-0.063478097	-0.094830579	-0.020360943	-0.022447515	0.281607194	0.277791599	0.10632094	0.148432824	0.440785582	0.323806678	0.408189132	0.125594651	0.19427509	0.167497859
Stimulaotry	HMGB1	0.044028231	0.016961106	-0.062571665	-0.071126857	0.520216625	0.105041744	0.255230455	0.237355388	0.414115584	0.278112151	0.008540751	-0.001671642	0.25509138	0.228393754
Stimulaotry	IL1A	-0.042779291	0.12755665	-0.06750414	-0.141389377	0.105280675	0.23758099	0.029207544	0.197987759	0.334010856	0.104821602	0.056979935	0.106224129	0.134922711	0.025788196
Stimulaotry	CX3CL1	-0.01808816	0.226491198	-0.044288355	-0.166195869	0.268917063	-0.289879212	-0.144835245	0.433963412	0.079469784	0.178755624	0.251664217	-0.008447395	0.082741101	0.099831779
Stimulaotry	IFNA1	-0.091278542	0.071832383	0.0312448	-0.107837839	0.087736166	-0.046003273	-0.034253402	0.183718441	-0.220395138	-0.050618636	0.073615218	-0.122286015	0.017005422	-0.059206914
Stimulaotry	IFNA2	-0.042891056	-0.027735382	0.029530095	-0.095081126	0.140283714	-0.218630569	-0.175849368	-0.094341902	-0.075718096	-0.066512984	-0.001814172	-0.032473041	0.008484076	0.049190145

© 2023. Feng D et al. Published online at http://www.aginganddisease.org/EN/10.14336/AD.2023.1126

SUPPLEMENTARY DATA

Stimulaotry	CD40	-0.081386172	0.045882004	0.048092507	-0.206323196	-0.19278217	0.460149175	0.221057582	-0.227934688	0.190777388	0.40690678	0.285368689	0.193570466	0.294501379	-0.063507064
Stimulaotry	ENTPD1	-0.162266605	-0.153135854	-0.023637317	-0.180355315	0.177902216	0.423291425	0.24962803	-0.138559753	0.338353949	0.333334748	0.111392006	0.040907228	0.298559245	0.117060801
Stimulaotry	TLR4	-0.198657545	-0.11793118	0.004399383	-0.030221507	0.064375764	-0.171435582	-0.112482084	-0.116679041	0.264421923	0.191074572	-0.033472887	-0.027003728	0.223354066	0.037478294
Stimulaotry	ICAM1	0.159267912	0.101741135	0.053358389	0.018985327	0.350785409	0.518386997	0.304910186	0.65811815	0.43531266	0.575349628	0.49787234	0.523091025	0.317279877	0.2040702
Stimulaotry	ITGB2	-0.031216989	-0.123185028	-0.139445429	-0.132337635	-0.061544528	0.481137118	0.372135672	0.498271623	0.449394107	0.445853617	0.264878883	0.3094469	0.263310009	0.121448877
Stimulaotry	TNFRSF18	0.082986157	0.143173491	-0.038644656	-0.169601067	-0.170043479	0.316727539	0.072484025	0.50930492	0.262350197	0.323607334	0.350920358	0.190272747	0.227241341	0.003604667
Stimulaotry	CXCL10	-0.095188925	-0.202976783	-0.175165998	-0.000407233	-0.145375434	0.58308597	0.367137034	0.459218782	0.237704576	0.235958103	0.015149903	0.063229692	0.140524012	0.104141432
Stimulaotry	CXCL9	-0.103193807	-0.253689183	-0.157200877	-0.067618004	-0.229126955	0.541939219	0.382984313	0.48252605	0.329213812	0.308552404	0.067613911	0.10579966	0.146041389	0.069397638
Stimulaotry	PRF1	-0.094620432	-0.190073894	-0.028645061	-0.135038407	-0.23124047	0.571518519	0.37454237	0.538673163	0.413636175	0.325470655	0.350223868	0.070815926	0.209956694	0.053415254
Stimulaotry	GZMA	-0.161022375	-0.22859932	-0.046679919	-0.192377032	-0.330741474	0.497028772	0.365422828	0.469575063	0.271522211	0.315115346	0.036310893	0.088078191	0.155810855	0.000799856
Stimulaotry	CCL5	-0.154959416	-0.162099862	-0.115973191	-0.201675419	-0.254038778	0.515770352	0.346454009	0.561353968	0.434171798	0.35861368	0.146618116	0.160120807	0.211550417	0.138106415
Stimulaotry	CD70	-0.194327104	-0.183830191	-0.15107749	-0.144183875	-0.017597873	0.538774914	0.31911009	0.487013703	0.263591534	0.437923162	0.205994289	0.300094147	0.108007264	-0.021707235
Stimulaotry	TNFRSF4	0.034042959	-0.090929356	-0.0794361	-0.189434418	0.13478981	0.576375955	0.381830879	0.466325029	0.110452373	0.231684812	0.178744589	-0.075158575	0.233153664	0.069890571
Stimulaotry	CD80	-0.185676796	-0.179306501	-0.130210651	-0.205969524	0.237714771	0.5301421	0.352737516	0.44102873	0.34573172	0.323794121	0.074597155	0.151785721	0.266349899	0.08388825
Stimulaotry	IL2RA	-0.042452366	-0.120664298	-0.064456136	-0.170914633	0.084336766	0.520523844	0.292630345	0.32946643	0.401465979	0.377598123	0.328171401	0.199576716	0.353203001	0.071146114
Stimulaotry	TNFRSF9	-0.061719331	-0.15108479	-0.049687181	-0.135402428	0.181619723	0.421062583	0.297671227	0.480608305	0.373150675	0.423892581	0.168843281	0.232026882	0.269245333	0.100806695
**TYPE**	**GENE**	**LUAD**	**LUSC**	**CESC**	**PRAD**	**ACC**	**GBMLGG**	**LGG**	**UVM**	**KICH**	**KIPAN**	**GBM**	**KIRC**	**KIRP**	**BLCA**
Stimulaotry	TNFRSF14	-0.058452308	-0.11965326	0.069470317	0.152852924	0.163930332	0.537516634	0.299180904	0.51630964	0.089072122	0.092831564	0.310996296	-0.068540442	-0.109891816	-0.058394963
Stimulaotry	IL1B	-0.036020584	0.038500226	0.025659809	-0.12971269	-0.011806624	0.134122843	-0.04294152	0.351915583	0.234449213	0.272011785	0.199860613	0.159370201	0.179500952	0.02537342
Stimulaotry	BTN3A1	-0.077675223	-0.180241827	0.133705724	-0.063498696	0.363583785	0.436206109	0.396730116	0.261311976	0.513166822	0.348263044	0.046095506	0.04806327	0.277792786	0.136131247
Stimulaotry	BTN3A2	-0.045708787	-0.119403696	0.048039695	-0.086792283	0.360218729	0.479599429	0.412887614	0.378191201	0.39676443	0.359473303	0.167449738	0.065168315	0.306114801	0.082668032

0 < absolute value of r < 0.3 | **0.3 < absolute value of r < 0.5** | **absolute value of r > 0.5** | **p > 0.05**

© 2023. Feng D et al. Published online at http://www.aginganddisease.org/EN/10.14336/AD.2023.1126
